# Daily work variability in falls prevention of hospitalized patients: nursing team’s perception

**DOI:** 10.1186/s12913-023-09956-w

**Published:** 2023-08-31

**Authors:** Deise Vacario de Quadros, Priscila Wachs, Ana Maria Müller de Magalhães, Isis Marques Severo, Juliana Petri Tavares, Daiane Dal Pai

**Affiliations:** 1 Hospital de Clínicas de Porto Alegre, Porto Alegre, Rio Grande Do Sul Brazil; 2 Federal University of Rio Grande Do Sul, Porto Alegre, Rio Grande Do Sul Brazil; 3 Pontifical Catholic University of Rio Grande Do Sul, Porto Alegre, Rio Grande Do Sul Brazil

**Keywords:** Nursing, Accidental falls, Patient safety, Workflow, Complexity, Socio-technical systems

## Abstract

**Background:**

The identification of safety incidents and establishment of systematic methodologies in health services to reduce risks and provide quality care was implemented by The World Health Organization. These safety incidents allowed the visualization of a vast panorama, ranging from preventable incidents to adverse events with catastrophic outcomes. In this scenario, the issue of fall(s) is inserted, which, despite being a preventable event, can lead to several consequences for the patient, family, and the healthcare system, being the second cause of death by accidental injury worldwide, this study aims to identify the variability inherent in the daily work in fall prevention, the strategies used by professionals to deal with it and the opportunities for improvement of the management of work-as-imagined.

**Method:**

A mixed method approach was conducted, through process modeling and semi-structured interviews. The study was conducted in a public university hospital in southern Brazil. Study steps: modeling of the prescribed work, identification of falls, modeling of the daily work, and reflections on the gap between work-as-done and work-as-imagined. Medical records, management reports, notification records, protocols, and care procedures were consulted for modeling the work process, and semi-structured interviews were conducted with 21 Nursing professionals. The study was conducted between March 2019 and December 2020.

**Results:**

From July 2018 to July 2019, 447 falls occurred, 2.7% with moderate to severe injury. The variability occurred in the orientation of the companion and the assurance of the accompanied patient's de-ambulation. The professionals identified individual strategies to prevent falls, the importance of multi-professional work, learning with the work team, and the colleague’s expertise, as well as suggesting improvements in the physical environment.

**Conclusion:**

This study addressed the need for fall prevention in the hospital setting as one of the main adverse events that affect patients. Identifying the variability inherent to the work allows professionals to identify opportunities for improvement, understand the risks to which patients are subjected, and develop the perception of fall risk as a way to reduce the gap between work-as-imagined and work-as-done.

## Background

A fall is defined by the unintentional displacement of the body to a level below the initial position without the possibility of timely correction, compromising stability and aggregating many associated factors [1, 2]. It is an occurrence related to care rather than the underlying disease, which prolongs the patient's length of stay or results in a disability to the point of discharge [1].

This unwanted situation breaks with the quality of care, exposing healthcare institutions and their professionals. In addition, falls increase hospital costs [3], length of hospital stay, and predisposition to other falls due to the patient's fear of falling again [4, 5].

Falls represent a public health problem worldwide, being considered the second leading cause of death by accidental injury, with age as the main risk factor [1]. Thus, they represent a challenge to the professionals who care directly for the patients, the leaders who manage this care, and the organizations.

The planning of personnel and equipment resources tends to mitigate the risks to which patients are exposed. However, this strategy requires the engagement of professionals, focus on the work process, and patient orientation considering the professionals' knowledge of the patient's profile [5].

Identifying the risk factors for falls is the first and most crucial step in preventing falls [5], reinforcing the need for healthcare professionals to know predictive factors and the criteria for identifying and assessing patients through the risk prediction models [4].

Despite the prediction models helping identify patients with a higher risk of falling, there may be, on the part of the professional and the patient himself, a mismatch between the perceived risk of falls and the real risk, as well as in the measurement of this risk. Faced with the complexity of the outcome, the execution of the prescribed work does not always occur as planned [6].

In the gap between work-as-imagined (WAI) and work-as-done (WAD), there is considerable potential for variability represented by falls, facts that are justified by the presence of independent agents who act without the understanding of the whole, agents who adapt based on their experiences and due to the self-organization of systems, making it more likely to influence the behavior of the system than to control it [7]. Furthermore, these characteristics reinforce the hospital institutions as Complex Socio-technical Systems (CCS) in which the interaction of these elements does not present linearity, causing small changes in the work processes that may cause disproportionate results [8].

In CSS, such as hospital settings, it is necessary that the understanding of the gap between WAI, such as care protocols and standard operating procedures, and the WAD (work that occurs in practice) be analyzed to identify future interventions aimed at improving patient safety and quality of care by bringing WAI closer to WAD [9].

In order to understand the gap between WAI and WAD, the Functional Resonance Analysis Method (FRAM) was created. FRAM is a Resilience Engineering tool that makes it possible to analyze a work process´s variability that can influence the outcome of an event [10]. Focused on understanding the impact of this variability, FRAM allows work processes to be reconstructed, through modeling, comparing planned work with executed work. The study aims to identify the variability inherent in the daily work in fall prevention, the strategies used by professionals to deal with it, and the opportunities for improvement of the management of work-as-imagined. The research question was: What is the variability (WAI/WAD gap) of the activities involved in the occurrence of fall(s) in adult hospitalized patients?

### Patient safety and the fall

Patient safety is defined as the reduction, to the minimum acceptable, of the risk of unnecessary harm during health care [11]. Although unsafe acts in health could occur on a large scale, in the 90 s, in a publication by the Institute of Medicine, the issue of patient (un)safety gained worldwide repercussions through the disclosure of incidents that affected patients within health institutions. Since then, patient safety has started to be treated with relevance and institutions have been showing more, and more concern with their events and rethinking ways to work with them [12].

The World Health Organization, by identifying these safety incidents, has established systematic methodologies in health services to reduce risks and provide quality care. Furthermore, identifying safety incidents allowed the visualization of a wide panorama, ranging from preventable incidents to adverse events with catastrophic outcomes, such as death, for patients. In this scenario, the issue of fall(s) is inserted. Despite being a preventable event, it can lead to several consequences for the patient, family, and the healthcare system, being the second cause of death by accidental injury worldwide [13].

Identifying risk factors for falls in hospitalized patients facilitates more accurate measurement of the risk of falling and positively impacts patient safety [2]. The predictive risk factors for the event can be classified into intrinsic (patient-related) and/or extrinsic (environment- and work-related) [2, 4].

Among the intrinsic risk factors, disorientation/confusion, sleepiness, dizziness, agitation, hallucinations, delirium, cognitive impairment, age extremes, impaired mobility, and balance, altered visual acuity, frequent urination and/or diarrhea, history of falls, comorbidities, and medication effects stand out. The extrinsic risk factors related to the environment are slippery floors, inadequate lighting, and excessive furniture, among others, as well as work process factors such as the number of patients/nurses, high work demand, absence of a caregiver at the time of a fall, typology of units such as orthopedic, neurological and psychiatric units, the response time of the bell, and peak hours of activities [4, 5].

The next step after the identification of risk factors is the implementation of measures to minimize the possibility of falls through protocols or SOPs (Standard Operational Procedure), followed by improvements in communication between different professionals of the team who are in charge of the patient's hospitalization, as well as the continuous strengthening of the safety culture of an institution with the adaptation of specific needs [3]. Although SOPs are based on the identification of common factors for patients and show success in their implementation, it is necessary that risks are individualized, using information technology and tools [14] as a means to bring the WAI closer to the WAD.

Thus, in addition to prediction scales and SOPs, the identification of the role of the direct caregiver, represented by the nursing technician, the care administrator, in the figure of the nurse, and the decisions focused individually on the patient and their family (constituted by the multi-professional team) place a fall assessment and prevention program at the forefront of a hospital institution [14, 15].

### Functional resonance analysis method to analyze the variability of daily work

There is a noticeable distinction between the items that are part of WAI in the form of protocols and procedures (WAI) and the way the work is conceived in daily practice (WAD) [16]. In this sense, the gap that arises between the WAI and WAD can be explained by the variability of factors ranging from a need to set priorities within an environment with high complexity to an unfavorable work environment and even by human conditions [10].

The search for the reduction of the WAI/WAD gap stimulates the development of actions, making health institutions establish quality and safety standards, minimizing errors, and working with predetermined standards in order to achieve international patient safety goals, specifically in this context, reducing the variability that predisposes to the instances represented by the falls [17].

The concept of WAD and WAI presented so far has, as its theoretical basis, Resilience Engineering (RE). The RE emerges as a paradigm for security management, especially for CSS, which seeks to measure, assess, and improve the resilience of a system [18]. From the RE perspective, the resilience of a system is defined as "the intrinsic ability of a system to adapt its functioning, before, during, or after some change or disorder, in order to maintain necessary operations under expected and unexpected conditions" [19]. Under the same optics of RE, the Functional Resonance Analysis Method (FRAM) arises.

FRAM is a method used to describe the activities of CSS, capturing the daily work´s variability (actual or potential). FRAM is based on four principles: (i) equivalence – which indicates that both success and failure arise from the same processes; (ii) performance adjustment—people continually adjust to account for the contextual conditions at that given time; (iii) emergent phenomena—outcomes, in SSC, cannot be explained with a linear cause-and-effect relationship; (iv) functional resonance—can be used to understand the interactions and non-linear outcomes [10].

**Fig. 1:**
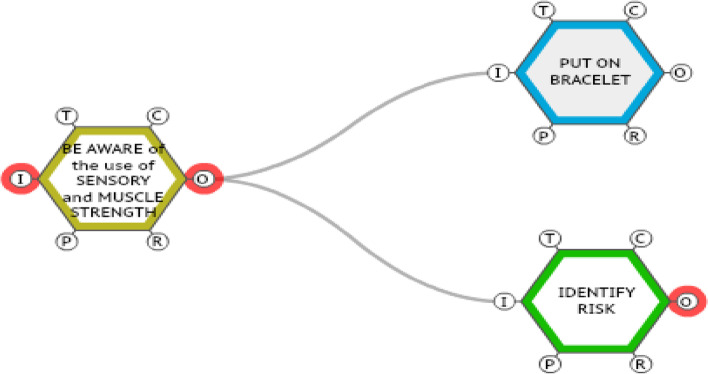
Functions and Couplings. Source: The authors, Porto Alegre, RS, Brazil, 2022

FRAM identifies and describes the main functions of the analyzed system, considering its potential (or real) variability. For each function identified in the system, the following aspects can be described: (a) Input—activates or initiates the function; (b) Output – the result of the function; (c) Precondition—state or conditions to initiate the function; (d) Resource—required or consumed during the realization of the function; (e) Time—temporal characteristic of the function; (f) Control—supervises or regulates the function [10]. The different functions interact through couplings (Fig. 1) when an output of one function is the input, precondition, resource, time, or control of another. Moreover, it is in this coupling that the variability of the output of a function ends up impacting the performance of the functions coupled to it.

Figure 1 represents a cutout of the process of measures to prevent falls in adult inpatients and highlights the coupling of the functions < attend to the use of drugs that alter sensory and muscle strength > , < identify risk > , and < put on bracelet >. The variability, represented by a symbol similar to a sine (or wave), in the output of the function < attempt to use drugs that alter sensory and muscle strength > , in terms of precision (e.g., not perceiving possible alteration of sensory) or in terms of time (e.g., perceiving it too late), has repercussions on the triggering of the functions coupled to it downstream. These functions are critical in the process of preventing falls.

FRAM has been gaining space to analyze CSS, such as health services. Regarding studies in healthcare that used FRAM to analyze the variability in the daily work and the GAP between WAI and WAD, the following studies were identified: (i) modeling interactions between procedures (WAI) and resilience skills, enabling, i.e., the identification of underspecified rules and resilience skills. This study was developed in an emergency department [20]; (ii) analyzing the everyday work of the flow of the critically ill patient between the emergency department and the Intensive Care Unit. This study presents four examples of problems on the analyzed flow concerning the FRAM function and the variability resulting from the problem. After that, the study presents design and assessment of countermeasures [21]; (iii) understanding WAD and approximate WAI to that performed during the process of guideline implementation in ICUs [16]; (iv) demonstrating the difference between WAI and WAD about falls and delirium in older inpatients [22]; and in a part of the same research project as this study, analyzing falls of adults hospitalized patients and their repercussions on Nursing workers as a second victim [23]. The first three studies were developed in specific departments of healthcare institutions and used FRAM to understand the variability of the work-as-done and, in some extension, the strategies used by professionals to deal with it [20] and the opportunities for improvement of the work-as-imagined [16, 20, 21]. The last two studies focused on falls and used FRAM to analyze them: one on understanding the gap between WAI and WAD and the impact on the Clinical Decision Support System [22]; the other with a second victim approach.

## Methods

This study aims to identify the variability inherent in the daily work in fall prevention, the strategies used by professionals to deal with it, and the opportunities for improvement of the management of work-as-imagined. The methodological approach used to achieve the aim of the study was a mixed method approach, which amplifies the analytical possibilities [24]. The mixed method approach has been adopted in nursing studies to address complex research questions [25].

The study was conducted in a public, general, and university hospital with a capacity of 843 beds. This hospital is located in the South of Brazil and is considered a reference center for assistance, professional training, and knowledge generation. It has an occupancy rate of 125,396 patients/day, 42,847 surgeries/year, and 12,786 hospitalizations/year.

With specific regard to incidents involving patient fall, since 2012, the institution has had a Multi-professional Commission on Falls under the coordination of two nurses. The committee meets weekly with representatives from all services of the institution, analyzes the occurrence of falls, propose improvements, and monitors, through indicators, the rates of falls in the institution.

### Study steps

**Fig. 2:**
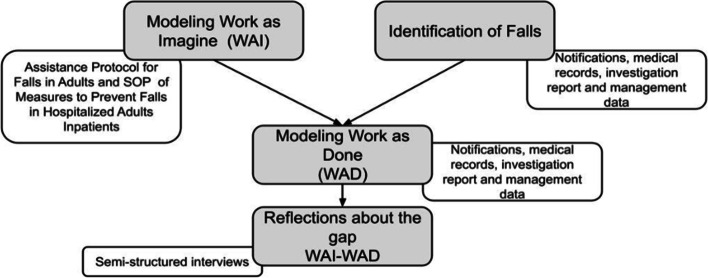
Study steps. Source: The authors, Porto Alegre, RS, Brazil, 2022

Figure 2 shows the steps taken to develop this study: modeling (WAI); identifying falls; modeling daily work (instantiations of the identified falls) (WAD), and, finally, reflections on the gap between WAI and WAD.

### Step 1—modeling the work-as-imagined

The first stage proposes to portray the work-as-imagined. Data were collected in August 2019 from document analysis, as the care protocol for falls in adults and the SOP for measures to prevent falls in hospitalized adult patients.

Data analysis was performed using the Content Analysis Technique, following the three systematic phases: pre-analysis, material exploration, and treatment of results, inference and interpretation [26]. The third phase (treatment of results, inference, and interpretation) was conducted based on the Handbook for FRAM modeling and encompasses [10]: identification and description of the main functions (and aspects) of the system, characterization of the potential variability of each function, aggregation of the variability (couplings). After the data analysis, the findings were inserted in FRAM Model Visualizer Software, and the FRAM model image was obtained.

### Step 2—identification of falls

To identify the falls, the population was composed of the total of hospitalized adult patients who had a fall (*N* = 447) stratified into clinical and surgical inpatient units (*N* = 242). The sample was made up of patients who had moderate harm to death (*N* = 12). Patients who had moderate to fatal falls represent a smaller number, however, it is possible to extrapolate the analysis for falls without injury to mild injury, since they occur in the same way, although with less repercussion to the patients.

Data collection in August 2019 for the identification of falls was performed in medical records, notifications, and investigations report and management data. Analysis of the data allowed the severity of the damage to be identified, constituting the second stage of the study. The period consulted was from July 2018 to July 2019.

### Step 3—modeling the daily work

The data collected and analyzed in the previous steps were considered to perform this step. Thus, the third step consisted in modeling the real work (falls identified in the previous step), considering the instantiations of daily work.

The data sources were the Management Information System, the Strategic and Operational Management System, and information from medical records. Data were collected from August to September 2019. For such modeling, we took, as a starting point, the modeling of the prescribed work, identifying the variability that occurred in the functions identified previously (WAI modeling) and the functions that emerged from the analyzed event. It is worth noting that the same data analysis process used in the first step was undertaken: The Content Analysis Technique of the collected documents.

### Step 4—Reflection on the gap between work-as-imagined and work-as-done

Nursing professionals who worked in inpatient units were the population of this study stage, totaling 153 professionals (*N* = 153). The study participants were invited among those who worked in the units and in the shifts in which the falls occurred. To maintain anonymity, the participants were coded as NURS for nurse and NT for nursing technician, adding the number of the interview. They were asked to identify the care prescribed and how the work occurred on a daily basis to implement the protocol that identifies the risk of the patient falling.

Data were collected from March to May 2019 using a semi-structured interview technique with an average duration of 35 min. One nurse and two nursing technicians were invited to participate for each shift in which a fall had occurred. The interviews were closed when data saturation occurred, totalizing 21 nursing professionals (*N* = 21). Data collection began with the study presentation and Participant´s Informed Consent (PIC) reading. After consent, the participant was contextualized about the previous stages of the study through a modeled event, the use of the modeling tool (FRAM), the variability that occurred´, and its implications on the quality of care. Next, the interview script started with the fall WAI and WAD modeling for presentation (including a discussion about the functions, the variability of the functions, and the gap between WAI and WAD), followed by the questions: What are the difficulties identified by the professional for the patient to adhere to the institutional protocol? How is it possible to minimize the occurrence of the event?

As in the previous steps, the last step used the Content Analysis technique for data analysis [26]. The analysis procedures aim to explore the manifest content of the messages so that it is possible to identify the units of record, which were coded and classified so that they can answer the guiding questions raised in the interviews.

### Ethical considerations

This research was registered in *Plataforma Brasil* under CAEE no. 35069714.7.0000.5327 and approved by the Research Ethics Committee (REC) under Opinion no. 2.554.758. The researchers committed to maintaining the information secrecy and confidentiality at all stages, meeting the aspects contemplated in Resolution No. 466/12 of the National Research Ethics Committee (Brasil, 2012). A Term of Commitment for the Use of Institutional Data was filled out for the use of the hospital databases, ensuring the use of the information collected for research purposes only. The participants of the interview were given the PIC. The speeches were recorded on digital audio, later transcribed without the use of software, and kept under the custody of the principal researcher for five years as recommended by the National Research Ethics Council (CONEP) (Brasil, 2012).

### Rigour

The guarantee of methodological rigor in qualitative research occurs through the path chosen by the authors in which they can justify the decisions made and ensure that the data collection procedures and the instruments used to guarantee the ethics and transparency necessary for research. This care enables the results of a qualitative study to have internal and external consistency, ensuring the reliability of the analyses performed [26, 27].

To ensure credibility, reliability, and findings in the design of this study, a validation of the results found in the prescribed process modeling step was performed through an interview with the professionals. Before data collection, a pilot interview was conducted with a professional who was not part of the study to calibrate the questioning sequence. In addition, the principal investigator held frequent meetings with members of the research team who have expertise in the field, which enabled a consensus regarding the interpretation and categorization of the data. In addition, the principal investigator is a nurse with experience in the work processes of the study's focus institution and in conducting semi-structured interviews.

To ensure transparency, the Consolidated Criteria for Reporting Qualitative Research (COREQ) recommendations were followed [28].

## Results

### Modeling the work-as-imagined

**Fig. 3:**
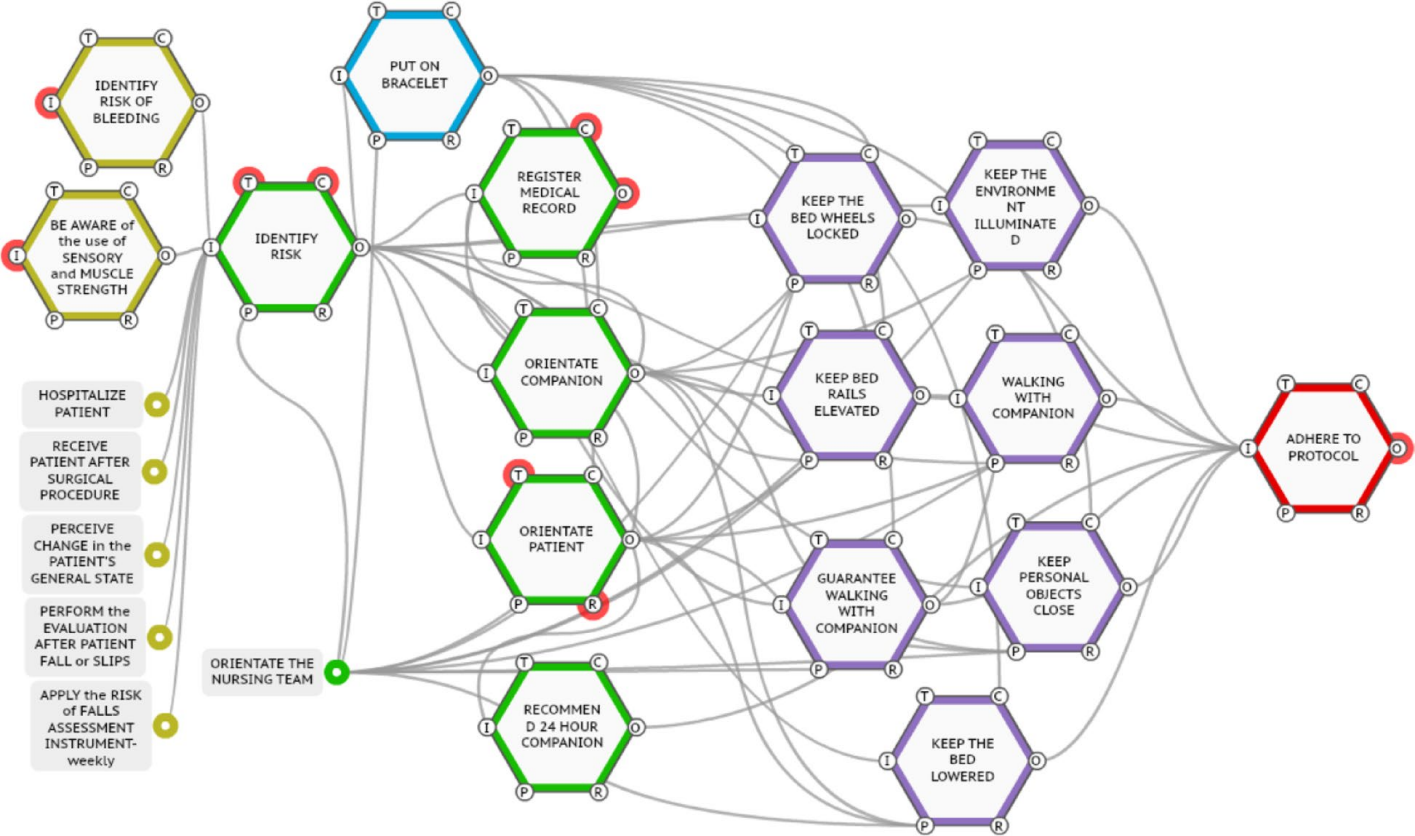
Work-as-imagined. Source: Quadros et al. (2022) [23]

From the care protocol for falls in adults and the SOP for measures to prevent falls in adult inpatients, it was possible to model the prescribed process. The prescribed process was modeled with the help of a software called FRAM Model Visualizer and is represented in Fig. 3.

A total of 22 functions related to the process of preventing falls in adult inpatients were identified. The functions represented in yellow indicate functions that initiate the modeling. The seven functions that initiate the modeling (in yellow) may occur in different moments: when the patient is admitted; when receiving the patient after the surgical procedure; when noticing a change in the patient's general state; When assessing after the patient falls or slips; when applying the assessment tool for fall risk (weekly) and contemplating some comorbidities of the patients that lead to individual risks of falling, such as the risk of bleeding and the use of drugs that alter the sensory and muscle strength.

From the function that identifies the risk (< identify risk >) and visually signals the professionals (< put on bracelet >), five other functions are inserted. They are related to the orientations that are necessary for the Nursing team, the patient, and the caregiver and are defined after the identification of risk by: medical record; orientation of the caregiver; orientation of the Nursing team; orientation of the patient, and recommendation of a full-time caregiver. These functions are represented in Fig. 3, in green.

From the orientation given to the Nursing team (< orientate the nursing team >) and the identification of risk (< identify risk >), seven other functions, represented in purple, are added. These functions deal with actions to be implemented in the work process: < keep the bed wheels locked > ; < keep the environment illuminated > ; < keep the bed rails elevated > ; < keep personal objects close by > ; < keep the bed lowered > ; < walking with companion > and < guarantee walking with companion > .

The functions medical record (< medical record >) and implementation of care (seven purple-colored functions in Fig. 3) occur simultaneously. The boundary function of this modeling is < adhere to protocol > . The output variability of all functions upstream of this one reflects in not fully adhering to the protocol, potentially triggering a crash event.

The representation of the functions by colors elucidates the different moments in which the SOP is implemented by grouping actions: i) yellow: conditions for the application of the fall risk scale; ii) green: orientations and registers; iii) blue: visual signaling of patients who have the risk of falling to professionals; iv) purple: prescribed care to prevent falling; v) red: modeling limit.

Critical functions relate to patients, companions, and the Nursing team orientation. This identification can be evidenced by the number of couplings perceived downstream of the functions. Functions with more upstream couplings have a greater chance of receiving variabilities, such as the function < identify risk > , with eight upstream couplings, and the function < adhere to protocol > , also with eight upstream couplings. In contrast, functions with a greater number of downstream couplings may increase the variability repercussions of their output.

Regarding the design of the final process, it is entirely human-dependent, and its functions are characterized in the FRAM Model Visualizer software as human. The nursing professional performs the beginning of the process, the evaluation of the risk score, and the orientation of the Nursing team, and all other functions are performed by the Nursing team.

### Identification of falls with moderate to severe injury

As identified in the institutional notifications, 447 falls were notified from July 1^st^, 2018, to July 31^st^, 2019. Among those falls, 242 (54.1%) were related to the clinical and surgical inpatient units, and among them, 12 (2.7%) represent the falls with moderate to severe injury. There were no deaths resulting from falls in the analyzed period.

### Modeling the work-as-done

The 12 falls were analyzed from the prescribed work modeling (Fig. 3), being identified which functions presented variability. After this analysis, the falls were grouped into four instantiations.

The falls grouped in each instantiation presented variability in the same functions. Thus, in instantiation A, falls 1 and 11 presented real variability in the functions: < be aware of the use of sensory and muscle strength altering drugs > ; < perceive change in the patient's general state > ; < orientate companion > ; < recommend companion > ; < guarantee walking with companion > . Instantiation B, in turn, represents the falls 2, 3, and 12 in which there was variability in the functions: < perceive change in the patient's general state > ; < guarantee walking with companion > ; < identify risk > .

The highest number of falls is found in instantiation C, contemplating falls 4, 5, 6, 7, 8, and 9. Only two functions presented variability in instantiation C: < orientate companion > and < guarantee walking with companion > . Finally, instantiation D, from fall 10, presented the greatest number of functions with real variability. In this instantiation, seven functions presented variability: < identify risk > ; < identify bleeding risk > ; < put on bracelet > ; < be aware of the use of sensory and muscle strength altering drugs > ; < orientate companion > ; < orientate patient > ; < orientate the Nursing team > .

It is worth mentioning that some functions presented variability in more than one instance: (i) < guarantee walking with companion > presented variability in instantiations A, B, and C (falls 1, 2, 3, 4, 5, 6, 7, 8, 9, 11 and 12) and only fall 10 did not present real variability in this function; (ii) the function < orientate companion > presented variability in instantiations A, C, and D (falls 1, 4, 5, 6, 7, 8, 9, 10 and 11); (iii) < perceive change in the patient's general state > showed variability in the instantiations (falls 1, 2, 3, 11, and 12); (iv) < identify risk > showed variability in the instantiations B and D (falls 2, 3, 10, and 12) and the function < attend to the use of drugs that alter sensory and muscle strength > showed variability in the instantiations A and C (falls 1, 10, and 11).

**Fig. 4:**
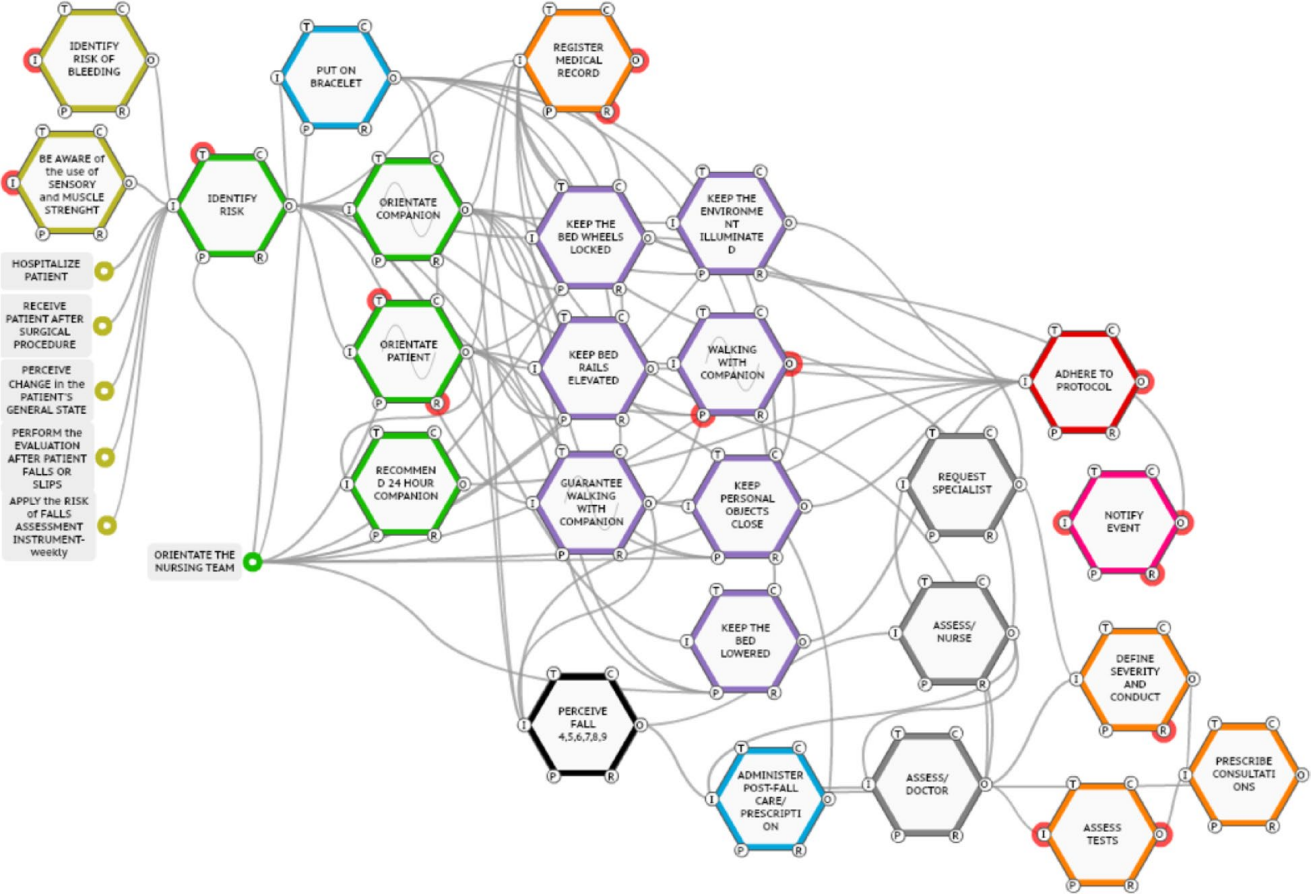
Modeling of instantiation C – work-as-done. Source: The authors, Porto Alegre, RS, Brazil, 2022

Figure 4 shows the modeling of one of the presented instantiations: the instantiation C.

All instantiations were modeled; however, we chose to present C since (i) the functions with variability in this instantiation (< orientate companion > and < guarantee walking with companion >) also presented real variability in other two instantiations; (ii) it presents the highest number of related falls (falls 4, 5, 6, 7, 8 and 9).

In addition to the 22 functions identified earlier, when the event occurs, other functions emerge. Thus, instantiation C presents 31 functions. The nine functions that emerged during or after the fall event are: < perceive fall > ; < assess nurse > ; < assess physician > ; < administer post-fall care/prescription > ; < solicit specialist > ; < assess exams > ; < define severity and conduct > ; < prescribe conduct > ; < notify event > . The new functions represented are: i) black color: the falls verified, corresponding to instantiation C; ii) gray color: the evaluations of the nurse, physician, and specialist; iii) light blue color: the new care needed to treat the fall; iv) orange color: the diagnostic exams and the indication of treatment, as well as the feasibility of necessary conducts depending on individual factors of each patient; v) pink color: the need to notify the adverse event.

In addition to the functions that were incorporated and now constitute work-as-done, preexisting functions, such as < medical record > and < guidance to companion > , received other couplings. For example, in the function of register in medical record, upstream, there was an increase from three to eight couplings, and in the accompanying person orientation, the variation was downstream with the incorporation of one more coupling.

### Reflections on the gap between work-as imagined and work-as-done

The reflections on the gap between WAI and WAD occurred during the fourth stage of the study in interviews with professionals. Thus, from the modeled falls, a fall was presented to the study participants to understand the difficulties identified for the effectiveness of the institutional protocol.

“(...)I lower the bed, explain why, reinforce the issue of having to have the bars up, which is something that patients have a lot of resistance, they don't like to have the bars up, even with the risk of falling, because I believe they feel trapped, with their freedom limited. I also put myself in their place (...) sometimes, we end up making the bars more flexible, which favors a fall.” (NURS 2)

“(...)she was wearing a bracelet, but she asked for the grid to be lowered, but the patient can't ask, she was an elderly woman (...), she wanted to be mobile.” (NURS 4)

Professionals' strategies used to account for variability related to patients' attitudes (orientate companion > , < recommend companion > , < guarantee walking with companion >).

“the bells have a bad wire, (...) a short wire, so we tie a compress to make it longer for the patient.” (NURS 5)

“(...)by the protocol, most of the care that we prescribe or that is in the protocol, we perform, but the end result is not always as expected, (…) sometimes we prescribe to request the presence of a family member, but the family member, does not come or comes, but does not pay attention and sleeps (...), but I think that, no matter how much we prescribe, there is this distance between the real and the prescribed (...) it is absurd, but we count on the neighbor's companion to provide support.” (NURS 6)

“(...)if there is no companion, what is the need for restraint, if you check with the medical team if there is a need for restraint. If you try to enter the room more, visit the patient more, also try to review if you can have a companion if the patient is alone.” (NT13)

“(...)or they orient the patients that they can walk pushing the IV drip stand and then it weighs, turns and the patient falls, sometimes there are two or three pumps in the stand, they also have to be educated for this.” (NT 12)

Sometimes the daily doer needs to find individual strategies to ensure patient safety, which sometimes ends up being incorporated by other colleagues. How these strategies are incorporated into the work process and disseminated among the teams values the professional when seeing an idea disseminated and encourages others to seek innovations. However, it must be considered that safety systems should be designed to help professionals carry out the work, avoiding shortcuts.

“I try to do what I call a crib protector, which is to take the two transfers of the patient and put one on each side of the bed, which makes falling more difficult, besides containing (...), most of them are already adhering to my idea because the beds are comfortable, but they are inappropriate (...) in winter, they stay uncovered a lot (...) I make a hut for them and I try to go to the room more often (...).” (NT1)

“(...)leave only his chair and don't leave another chair on the way back, take care of the slippers because, sometimes, when he gets up, he puts his feet together and ends up falling down.” (NT14)

Thus, the Nursing team becomes vigilant to the vulnerabilities of hospitalization. In this vigilance, nurses and nursing technicians should share the responsibilities regarding the risk of falling with the multi-professional team, as shown in the following excerpt.

“(...)the physical therapists (...) take the patients to walk in the corridor and don't orient them that they can't walk alone (...). The doctors, when they go to check on the patients, lower the bars and don't lift them, they go to do some procedure and also leave the beds high. Collection personnel... I think it is something kind of general (...).” (NT12)

The professionals identify that the SOP implementation does not guarantee the elimination of falls. They use their experiences and expertise to glimpse other latent situations that can contribute to the occurrence of falls once the protocol cannot cover all the possible situations that can result in the variability represented by falls.

“(...)I will take off the socks and put a sheet on the floor because, with the sock, he will slide with this wool sock (...) we are protecting the patient.” (NT4)

In addition, the professionals report the importance of shared work through a cohesive team, with previously built bonds, in favor of a common goal. Boosting the final result and providing opportunities for collective learning.

“(...)when there is a fixed person in the team, who is here every day, (...) the same person, we start to talk as a team and this flows very well. So, now, for example, when we are rotating more, when we don't have a fixed person, these problems increase.” (NTF7)

Still, the professionals interviewed brought suggestions to reinforce the patient's understanding of the risk of falling, revealing the need for greater awareness. That is a way to involve the patients in their care.

“(…)also, besides a little film, a little folder, because, if he doesn't understand, look there, (…), but it's a little thing that some scenes, if he sees that film or reads that there, something, he will record (…) explaining which are the risks of falling and such, what he should do to avoid (…).” (NT6)

Suggestions for improvement are also made regarding the environment, which implies the variabilities related to the Nursing team's competencies (< perceive change in patient's general condition > , < guarantee walking with companion > , < identify risk >).

“Safety bars on the shower stalls and something on the door (...) should have some locks.” (NT7)

“Low-light, light-on-the-way type, as soon as they are activated by movement.” (NURS6)

(...)the patients bring a lot of things, (...) like the TV, it gets in the way, wires on the floor, fan, it gets in the way a lot, (...) three extension cords in the room, it also causes them to fall.” (NT8)

Identifying opportunities for improvement in the environment shows the professional's commitment to qualify the assistance and bring the prescribed work closer to the real work.

## Discussion

During the studied period, 447 falls occurred. Although the 12 falls with moderate to severe injury may seem unrepresentative (2.7%), its analysis allows extrapolating the result to the other falls in the institution with less injury severity. This statement is based on the principle of equivalence [10], which states that situations with positive or negative outcomes arise from the same processes. Therefore, the reflection and the opportunities for improvement identified in the process will also reflect in reducing events with less severe outcomes, besides increasing the quality of care of events with positive outcomes.

Reflecting on the variability of the functions identified in the event of a fall, it can be seen that the function < guarantee walking with companion > , for example, presented variability in 11 of the 12 falls analyzed. Aligned with the principle of equivalence, it is understood that the variability of this function and the other functions with variability can also be present in situations where the fall does not occur but could occur.

Fall risk assessment makes it possible to identify patients who are likely to fall proactively. However, when the patient has prescribed care (falls 1, 4, 6, 9,11) and these risks are not foreseen, a careful look is not made to this process, and all patients with risk, whether low, medium, or high, are cared for in the same way [14].

Falls occur due to an accumulation of risk factors; thus, the importance of acting on known risk factors so that patients can benefit from prevention [15], offering the caregiver the opportunity to determine the type of care the patient should receive [14], so that the care prescribed can make sense to the caregiver, avoiding the trivialization of visual identification represented by the use of a fall bracelet.

It should also be noted that there are falls that can be anticipated, falls that cannot be anticipated, and accidental falls [29]. Thus, there are falls in which the assessment tool can be predictive (falls 1,4,6,9 and 11), those in which the tool does not identify the risk (falls 2 and 3), and the others in which the patient was not identified with the risk of falling (falls 7 and 10), but, due to problems related to the work process or the environment, they end up occurring [30].

Falls related to the work process and the environment are the second most common group after anticipatory falls [31]. These falls, although categorized differently, occur in the same way, and it is to these that the focus of prevention should be directed. In addition, however, there are falls 5, 8, and 12 in which, even if the risk was identified, care was not implemented to prevent it.

Facing a several falls that are not sensitive to the prediction instruments, the professionals' expertise is of great value in reducing them to make them avoidable. It is under this perspective that need to develop risk perception. Situations not foreseen in the protocol are supported by the tacit knowledge of the professionals and provide the reduction of the gap between the WAI and WAD [32]. Besides expertise, individual strategies, the sharing of ideas, and the construction of common goals, a cohesive work team and bond between members make it possible to reduce the variability that affects the gap between WAI and WAD.

Concerning the analysis of instantiation C (falls 4, 5, 6, 7, 8, and 9), variability can be seen in the function < orientate companion > , with nine functions implicated in the WAI and ten in the WAD. Throughout the patient's hospitalization, a large number of orientations are necessary, and these are performed by different professionals (nurses and Nursing technicians) in different shifts. In addition, the orientation is directed to multiple patients and family members/caregivers with different levels of understanding. This example illustrates the complexity inherent in hospitals, admittedly SSC, which have a many elements (human and equipment) that interact dynamically, which may result in a greater unexpected variability [33].

This variability sometimes impacts the fall as the final outcome, which is closely related to the quality of care and patient safety [14]. In addition to identifying that a large number of professionals perform orientation, once again reinforces the characteristics of CSS, i) large number of elements that interact dynamically and ii) diversity of elements [33], and additionally to this, the companion, further increasing the variability in this process, which is maintained by the uniqueness of patients and their companions [34], contributing to variability in the final outcome.

Another variability that permeates instantiation C concerns the function < guarantee walking with companion > , with five couplings in the prescribed work and six in the actual work. The variability represented by this function brings an additional challenge to professionals who need to guarantee this accompaniment in the patients' displacements, but in practice, due to the volume of work, it is not possible.

In addition, this prescription item goes against the care of the patient, who needs to mobilize himself in an increasingly earlier way, counting on the companion to help him, but is stimulated to become independent as a way to participate in his care actively. On the other hand, increasingly complex work processes, demanding attention and, consequently, a greater workload, mean that this wandering of patients is sometimes not accompanied by the professionals.

The effort of the professionals to contemplate the patients' needs, when they do not raise the bars, by request of the patients, or even when they tie a compress to make the bell wire longer and reduce the effort of the patients, in a certain way, has repercussions in the patient's safety when these flexibilities have a fall as an outcome. Furthermore, the empathy of professionals who are sensitized by noticing the absence of a companion, because they understand that there is no other way to occupy the patients' time and mind, sometimes provides the distance between the real work and the prescribed work. Thus, investment in the education of patients and companions is fundamental to ensure the safety and qualification of the assistance provided [5, 35], in addition to bringing them closer to the prescribed care.

To make patients partners in their care, it is important to involve them in the care plan, in the orientation of the risks involved, and in preventing falls [3]. A suggestion for improvement, presented by the study participants and aligned with these statements, is the presentation of educational videos and folders. Other equally important alternatives relate to the environment, revealing the need to strengthen safety barriers in the context of hospitalization.

By looking at the expertise of the professionals, suggestions for improvement in the work environment should be valued. The suggestions presented do not eliminate the risk of patients falling but constitute multimodal strategies of approach to the issue of falls, which value and involve the actors of care. Another way of valuing is the sharing of experiences and the dissemination of practices such as the use of "transfers" to be a bed protector and prevent the patient from falling through the bars. Actions such as this demonstrate the concern to ensure adherence to the fall protocol, but more than fulfilling tasks, participation in the work process and actions for patient safety are important [3]. Finally, one has to consider how much the valorization by the leadership stimulates the protagonism of the professionals and reduces the trivialization of all the quantitative falls that occur.

The complexity of the hospital environment provides that, to meet a range of specificities of the patients, it is proposed the incrementation of protocols, routines, and SOPs. In a certain way, this is the formal guarantee that the professionals seek to base their actions on; however, the sum of multiple individual protocols does not have real value when the same patient has multiple risks; that is, the sum of the parts does not translate the whole.

An elderly patient, with delirium, without prescribed fall prevention care, without a bracelet (fall 8), in which the fall leads to injury of the occipital region, or even a young adult patient who does not score in the fall risk assessment tool, using anticoagulant, which, by itself, provided a fall of great repercussion (orbit fracture, skull trauma, new surgery), reflects in the increase of hospitalization time [4], in hospital costs [3], on the image of the institution [5, 35] and the repercussions for caregivers. These falls have the potential to be catastrophic, and mitigating this injury is a big challenge. However, by looking at situations like this, you have to think about safer systems that can help caregivers.

Regarding the prescription and the fulfillment of the ideally planned work, the appeal to the nursing professionals is noticeable. There are specific items of orientation for the Nursing team who also implement them, but a multi-professional team sees the patient. Faced with a process as complex as falls, the involvement of communication between the different actors of care becomes fundamental [14], because successful fall management involves communication, SOPs, protocols, and teamwork [15].

Managing a multi-professional commission, such as the falls commission, is an important step to rethinking work processes. This strategy seeks to meet a challenge in the global scenario of patient safety represented by falls, but it is necessary to guarantee multi-professional care practices. The literature reports that doctors don't know how to identify where the fall scores are in the patient's records [22] or that the fall risk scale is not routinely discussed in all meetings of the different care teams involved in the care. These structural differences have repercussions in the differences of approach of the institution [36] and end up shaping behavior.

Investing in collaborative practices presupposes sharing work processes, not only the doing but also the planning, improving the continuity of care and patient safety [37]. To this end, organizations should invest in shared strategies as a way to improve work teams and patient benefits [38]. Involving the actors of care, understanding the difficulties, and testing suggestions provide a better involvement, promote healthy relationships, and allow the horizontal construction of the work. In addition,this discussion, when it occurs, provides a greater sense of belonging to the teams.

The speeches of the professionals can have recall implications, and the cross-sectional design can also be considered a limitation of the study, as well as the impossibility of generalization, considering the local context of the study. It is also necessary to ponder that the research developed addressed the vision of Nursing regarding falls. Considering the multi-professional dimension that this modality of risk requires, this is considered a limitation of the study. A complementary approach to the fall of adult hospitalized patients is presented in another study [23], which analyzed the same events considering the nursing worker as a second victim perspective.

Regarding the limitation of the study, it is sensitive to the context used because the modeling of the work process was performed from records of a specific context but does not invalidate the modeling in other environments, given the peculiarities of the scenario used. Considerations also shall be made regarding possible underreporting and patient outcomes. Furthermore, the interviewed professionals were not necessarily the same who were involved in the fall events analyzed, but they were involved in the falls in the hospital environment in general. Finally, the influence that may have been exerted by the researcher on the interviewees.

Understanding the variability in the occurrence of falls impacts the prevention and reduction of injuries to hospitalized patients, affecting safety and quality. Besides, professionals can identify the difficulties for patients to adhere to the protocol and the possibilities of mitigating the occurrence of falls.

## Conclusion

The use of WAI and WAD modeling allowed the identification of critical factors in the care process that contribute to the occurrence of falls. The findings of the study indicate that the variability inherent to the daily work in fall prevention is related to the assurance of accompanied walking, the orientation of the companion, verification of the change in the patient's general state, identification of risks, and the use of drugs that alter the sensory or muscle strength. To deal with the variability, the nursing professionals identified opportunities for improvement in the aspects related to understanding the patient's risk, which requires insistence on maintaining the bed rails, resources for comfort, and barriers to the unnecessary exit of the bed.

As for the variability related to the competencies of the Nursing team, the professionals interviewed listed the necessary improvements in the environment and structure. For example, a greater perception of the risk of falls can contribute to reducing the distance between WAI and WAD.

This study is innovative in the triangulation of the theme of falls, use of process modeling, and scenario of the work prescribed and performed. In addition, it identifies the importance of multi-professional work and allows the verification of strategies used in the care practice to contemplate the final objective of mitigating the repercussions of falls, even when the care protocol cannot predict all the situations that put the patient at risk of falling.

## Data Availability

The datasets generated and/or analysed during the current study are not publicly available due to they belong to a corresponding author’s master degree thesis. They will be available after July 2023 or under reasonable request to the corresponding author.

## Abbreviations

CCS: Complex Sociotechnical Systems
CONEP: National Research Ethics Council (CONEP)
COREQ: Consolidated Criteria for Reporting Qualitative Research
FRAM: Functional Resonance Analysis Method
NT: Nursing Technician
NURS: Nurse
RE: Resilience Engineering
REC: Research Ethics Committee
SOP: Standard Operational Procedure
WAD: Wor as done
WAI: Work as imagined
